# Is a single calibration for the TloadDback cognitive fatigue induction task reliable?

**DOI:** 10.3389/fpsyg.2025.1561819

**Published:** 2025-07-24

**Authors:** Jeromy Michael Hrabovecky, Xavier De Tiège, Chloé Samyn, Guillermo Borragán, Philippe Peigneux, Mélanie Strauss, Hichem Slama

**Affiliations:** ^1^Laboratoire de Neuroanatomie et Neuroimagerie Translationnelles (LN2T), UNI-ULB Neuroscience Institute, Université libre de Bruxelles (ULB), Brussels, Belgium; ^2^Neuropsychology and Functional Neuroimaging Research Unit (UR2NF), Centre for Research in Cognition and Neurosciences (CRCN), UNI-ULB Neuroscience Institute, Université Libre de Bruxelles (ULB), Brussels, Belgium; ^3^Department of Translational Neuroimaging, Hôpital Universitaire de Bruxelles (H.U.B.), CUB Hôpital Erasme, Université Libre de Bruxelles (ULB), Brussels, Belgium; ^4^Departments of Neurology, Psychiatry and Sleep Medicine, Hôpital Universitaire de Bruxelles (HUB), CUB Hôpital Erasme, Brussels, Belgium; ^5^Department of Neuropsychology and Speech Therapy, Hôpital Universitaire de Bruxelles (HUB), Université Libre de Bruxelles (ULB), Brussels, Belgium

**Keywords:** TloadDback task, cognitive fatigue, cognitive fatigue induction, cognitive capacity, methodology, calibration, reliability, validity

## Abstract

TloadDback task was introduced as a novel task for inducing cognitive fatigue by accounting for the cognitive capacity of each individual's processing time needs stimulus time duration (STD). The task is carried out on different days with calibration occurring on 1 day and fatigue induction occurring on another. The aim of this within subjects study is to assess the reliabilityThe of a single calibration. Fifty-one healthy participants (age = 32.2 +/– 13.45; sex *F* = 41) completed the TloadDback calibration phase on three different days at three different moments of the day (counterbalanced for morning, afternoon, and evening). Sleep quality, state fatigue and state sleepiness were considered as control variables. Comparisons across the 3 calibration sessions (χ^2^(2) = 34.1, *p* < 0.0001) showed a significant STD decrease (i.e., performance improvement) with the most salient between sessions 1 and 2 (*t* = 3.98, *p* = 0.0003^***^). However, the improvement occurred for only 2/3 of participants. STDs from the 3 calibrations were significantly correlated (α = 0.78). Differences in sleep quality, state fatigue and state sleepiness did not correlate with STD changes. Results indicate that a single calibration may not put all participants in their maximal cognitive load condition and that a second calibration could be more appropriate. Nonetheless, the fact that the 3 calibrations were significantly correlated and that 1/3 of participants did not vary between sessions 1 and 2 suggest that the measure is rather reliable and that a single calibration can be sufficient for placing participants in a close to maximal cognitive load condition for cognitive fatigue induction if a second calibration is not possible.

## 1 Introduction

Dual tasks are commonly used to assess cognitive capacity in outcome measures (Contemori et al., 2024) as well as in treatments for cognitive improvement (Einstad et al., 2021). The Time-Load Dual Back Task (TloadDback; Borragán et al., 2017b) was proposed as a novel and cost-effective task for inducing and measuring the impact of cognitive fatigue (CF). Particularly in the TloadDback, the dual task is used to measure cognitive capacity by introducing interference in cognitive control. According to the authors, the TloadDback reduces the time needed to induce fatigue in comparison to classically used extended stimulation approaches. This is achieved by determining, during a prior calibration session, the maximal cognitive load at which an individual can still efficiently perform the task. By calibrating the task according to an individual's cognitive capacity, the TloadDback is tailored for each participant. This can reduce the impact of inter-individual differences in cognitive capacities on fatigue induction, which is crucial when comparing, e.g., patients with brain disorders and healthy subjects.

The TloadDback is rooted in the framework of the Time-Based Resource-Sharing model, which is based on the assumption that attentional resources are limited and shared among cognitive functions and that cognitive load is impacted by the time available for processing simultaneous information (Barrouillet et al., 2004, 2007). Additionally, the Multiple Resource Theory (Wickens, 2002) posits that there is increased task interference in simultaneous processing when stimuli compete for resources from the same modality and attention channel. Therefore, cognitive load can be understood or interpreted as an individual's ability to process similar, simultaneous information under time pressure. By conceptualizing cognitive load as a function of time pressure, tasks can be adapted individually by calibrating the time available to process information based on task performance efficiency. Borragán et al. (2017b) went a step further by postulating that CF induction is affected by cognitive load. By continuously applying maximal cognitive load that can be processed efficiently, this should deplete the shared attentional resources. Therefore, CF can be induced by having an individual continuously performing at their individually pre-calibrated maximal cognitive load, predefined during the calibration session as the fastest presentation speed at which he/she is still able to perform the task efficiently.

Most prior studies have induced cognitive fatigue by manipulating the Time on Task (ToT) by using lengthy (up to hours long) cognitive tasks (Ackerman and Kanfer, 2009; Lim et al., 2010) or by increasing task demands (Cook et al., 2007; Shigihara et al., 2013). Using the TloadDback and its individually defined maximal cognitive load, it is possible to induce comparable conditions of cognitive load among participants with reduced ToT when compared to classical fatigue e paradigms (Borragán et al., 2016, 2017b). Since its creation, the TloadDback task has been used in a wide variety of studies investigating different aspects of CF, including fatigue type (Pickering et al., 2024), the effect of fatigue on cognition (Apreutesei and Cressman, 2024; Boaz-Curry, 2018; Smalle et al., 2021; Wierzchon and Derda, 2019), its neural underpinnings (Borragán et al., 2019), the presence of fatigue in multiple sclerosis (Borragán, 2018; Charonitis, 2021; Guillemin et al., 2025, 2022), the effects of fatigue on motor tasks (Holgado et al., 2023; Jacquet et al., 2021a; Salomone et al., 2021), fatigue reversal (Borragán et al., 2017a, 2018; Jacquet et al., 2021b; Wang et al., 2022), its impact on trust (Lopes et al., 2022), as well as methodological comparisons between fatigue induction procedures (O'Keeffe et al., 2020).

A crucial aspect of the TloadDback, and the motivation behind this study, lies in the calibration step of this task, which is supposed to be aimed at determining the maximal cognitive load to be used for a specific individual. Indeed, the task is typically carried out in 2 sessions: the calibration session and the fatigue induction session. These sessions are usually conducted on different days to avoid the effect of accumulated fatigue from successive practices. During the calibration session, participants complete a stepwise block format version of the task. At each block, the stimulus time duration (STD) is incrementally decreased, and stimuli are presented faster than in the previous block. This incremental decrease continues, block by block, until the participant's performance accuracy dips below 85%. The fastest speed at which the participant is still able to complete the task above 85% accuracy becomes the STD used to set the pace of the task during the fatigue induction session. Therefore, with this task, every participant should be pushed to their personal maximum processing speed (i.e., the maximum cognitive load for efficient processing under time pressure), which as explained above, allows for controlling for the impacts of interindividual differences in cognitive load during the fatigue induction session. This paradigm with individualized cognitive load calibration was not previously available in other versions of fatigue induction tasks.

As stated above, the reasoning behind why the two stages of the TloadDback are carried out on two different days is that it is an effort to avoid accumulated fatigue from the calibration phase from contaminating performance in the induction phase. However, attentional resources can vary throughout the day as well as from day to day (Brose et al., 2012; Matthews et al., 2017). Also, several factors can play a role in influencing attentional resources, such as learning and expertise (Abernethy et al., 2007; Keskin et al., 2023) as well as sleep, sleepiness and fatigue (Mullins et al., 2014; Spruyt et al., 2019; Tomei et al., 2006; Walker and Trick, 2018). Therefore, relying on a calibrated STD achieved on 1 day and after only one calibration session for inducing fatigue on another day might be disputed. The aim of the present study is to test for this potential bias in the TloadDback procedure by employing within-subjects, test-retest design. We asked participants to test and retest on three separate days the calibration session of the TloadDback and then compared the obtained STDs within subjects. The analyses also accounted for time of day, the previous night's sleep (number of hours, number of wakeups and subjective quality of sleep), as well as sleepiness and fatigue levels. If the calibrated STD is indeed a stable and reliable measure, there should be no significant differences between the three sessions.

## 2 Methods

### 2.1 Participants

Participants (*n* = 51; age = 32.2 +/– 13.45; sex *F* = 41) were recruited via word of mouth from the Brussels, Belgium area over the course of 2 months to participate in this test-retest validation study. Participants needed to have either no vision problem or corrected to normal vision and no difficulties with bimanual dexterity. Other exclusion criteria included the presence of neuropsychological/neurological disorders as well as the consumption of psychotropic and/or recreational drugs. The study was approved by the institutional ethics committee of HUB Hôpital Erasme (reference P2020/708) in accordance with the declaration of Helsinki. All participants provided a signed informed consent to participate.

### 2.2 Materials

#### 2.2.1 TloadDback

Measures of calibration speeds (i.e., STD) for the TloadDback task were gathered using a Windows laptop computer equipped with Matlab R2021b (MATLAB, 2021). The Matlab code for the TloadDback task is freely available at https://osf.io/ay6er/. Stimuli were presented on an external screen, positioned ~60 cm from the participant, using the Psychophysics Toolbox Extensions-3 (Brainard and Vision, 1997; Pelli, 1997; Kleiner et al., 2007).

##### 2.2.1.1 TloadDback procedure

The calibration session of the TloadDback is completed in two distinct steps, identical to Borragán et al. (2017b). As a familiarization to the task, participants are first exposed to familiarization blocks (30 items/block) for the number parity task and the 1-back letter task separately, and then to the combined letter-number task (60 items/block in alternation) at a slow presentation speed. The calibration itself consists of the combined letter-number task in which the STD decreases after each successfully completed block in a stepwise manner until performance accuracy consistently falls below 85%. The 85% cut-off is based on the research from the original authors (Borragán et al., 2017b), but since has been found to be the suggested level of performance for optimal learning (Wilson et al., 2019). For participants who are unable to attain 85% performance during the calibration session, they should be considered as not meeting inclusion criteria.

##### 2.2.1.2 Task familiarization

The STD employed during task familiarization was set at 1,500 ms. Stimuli were continuously presented, in white, in the center of a black screen.

For the number parity task, the numbers 1–9 were used, excluding the number 5 in order to have equal amounts of even and odd numbers as well as to avoid confusing a “5” with the letter “S.” Participants were asked to decide whether the number on the screen was either even or odd and to respond using the number pad keys 2 for even and 3 for odd with their right hand only.

The 1-back letter task consisted of the following letters: A, C, E, L, N, P, R, T, and U. Participants were asked to respond with their left hand on the space bar every time a letter directly repeated itself, thus relying on working memory updating.

The combined letter-number task contained 60 items (30 letters and 30 numbers displayed in alternation, e.g., A 3 N 2 N 4 R ….). Successful completion of the combined training task was set at 85% combined performance (Borragán et al., 2017b; Wilson et al., 2019), with numbers and letters weighted differently. As in Borragán et al. (2017b), the composite weighted tasks distributed 65% of performance accuracy to the letter task, since this task was deemed to have higher difficulty rooted in working memory updating. Upon completion of the combined familiarization block, performance was assessed. If a participant achieved 85% combined performance, they moved on to the final step of the calibration phase (see below). If 85% was not achieved, participants repeated another 60-item block at the 1,500 ms presentation speed and continued to do so until 85% performance was reached. No participants failed to reach 85% performance within 5 blocks.

##### 2.2.1.3 Calibration

Once participants completed the familiarization phases, the calibration phase of the TloadDback task began. Again, the calibration aimed at determining the fastest speed (shortest STD) at which a participant can successfully complete the combined letter-number task while maintaining 85% performance. This fastest speed was our dependent variable for the 3 sessions.

Participants were given a long pause (max 3 min.) prior to beginning this final step. Under advice from the authors of the original paper, each participant was advised the following to ensure maximum performance: (1) “If an error is committed, ignore the error and continue on in the task”; (2) “if attention lapses for any reason (“getting off-track”), begin anew with the current stimulus on the screen and continue on from there”; and (3) “pauses between blocks are self-controlled and should be handled in moderation (i.e., enough time to recuperate the resources spent during the block).” The first 2 counsels were advised to prevent continued errors from occurring due to an error-feedback loop as this would monopolize valuable attention. The third piece of advice was given to inform participants that they could take as much time as needed between blocks to recover from the demands of the task but not so much so that they lost the timing and motor components of the task. As stated above, the goal of the calibration was to determine the fastest speed at which a participant could successfully complete the TloadDback task; not to induce fatigue. Fatigue induction, not inherent to the present study, would normally have been accomplished at another time using a variation of this task that would be calibrated to run at the fastest calibration speed.

The calibration began at 1,400 ms in the same format, letter then number in alternating fashion. Each block contained 60 items, as in the familiarization block (30 letters and 30 numbers). Performance was weighted in the same way (65% letters and 35% numbers), and success of an individual block was calculated as a combined 85% accuracy. With each successful block completion, 100 ms were deducted from the previous STD, and the ensuing block was presented at the updated faster speed. The calibration continued in this stepwise block format until performance dipped below 85%. To be certain that the previously successfully completed speed was an accurate performance, a full block was repeated at the speed of the previous 85% success rate. This verification continued until three 85% performance levels were found for the same speed.

Participants participated in 3 different calibration sessions of the TloadDback task. In order to control for possible impacts on performance due to different moments of the day, all participants completed the calibration phase at three different times of day (morning, afternoon and night). The times of day were counterbalanced for all participants.

#### 2.2.2 Control factors: sleep, sleepiness and fatigue

As the calibration phase of the TloadDback is a complex working memory dual task that relies on divided and sustained attention, we decided to control for natural factors that can influence attentional resources throughout the day: quality of sleep, sleepiness, and fatigue. The aim was to account for factors that could possibly impact performance during the calibration sessions. If calibrations were found to fluctuate in kind with the measures taken, this could have negative impacts on the calibration-fatigue induction procedure for the TloadDback.

Participants were asked to complete 3 questionnaires at the beginning of each calibration session. The St. Mary's Sleep Quality Questionnaire (Ellis et al., 1981) was used to assess the previous night's sleep. In particular, the total number of hours of sleep, the number of wakeups during the night and the overall subjective assessment of sleep quality were used. The Karolinska Sleepiness Scale (KSS) was used to measure a subjective level of sleepiness prior to performing the task (Åkerstedt and Gillberg, 1990). Fatigue was also measured and controlled using the Visual Analog Scale of fatigue (VASf) and was also completed at the beginning of each calibration session (Lee et al., 1991).

### 2.3 Statistics

Data were analyzed with JASP (Version 0.18.1) and Jamovi (Version 2.3.18). Parametric statistics were carried out for all normally distributed data. Non-parametric statistics were used if data violated normality or sphericity tests. *P-values* were corrected for multiple comparisons using Bonferroni corrections. For 3 comparisons, α significance threshold was adjusted to 0.0167.

## 3 Results

### 3.1 Comparing STD calibrations across sessions

**Figure 1:**
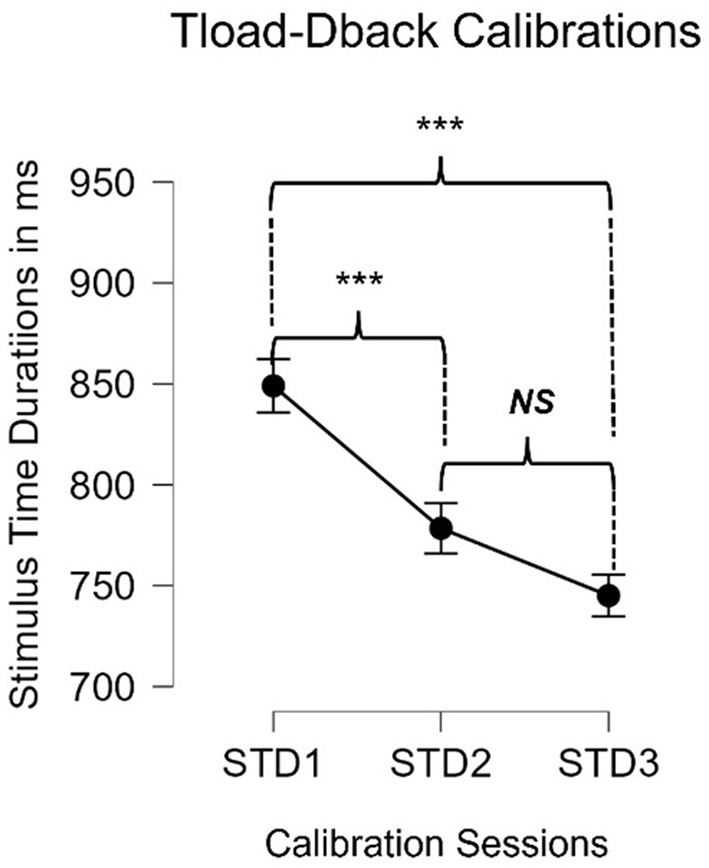
Mean values of the STDs of the TloadDback in milliseconds for each of the 3 Calibration Sessions. For *p-values* < 0.05 (*), < 0.01 (**), and < 0.001 (***).

Using Friedman's Non-parametric Repeated Measures ANOVA and controlling for multiple comparisons, we found an overall significant difference across the 3 training sessions (χ^2^(2) = 34.1, *p* < 0.0001^***^). Durbin-Conover pairwise comparisons revealed that the mean STDs for calibration session 1 was significantly slower than the mean STDs for calibration 2 (*t* = 3.98, *p* = 0.0003^***^). A similar finding was shown between calibration sessions 1 and 3 (*t* = 5.72, *p* < 0.0001^***^). The mean STDs for calibration sessions 2 and 3 did not reach significance (*t* = 1.73, *p* = 0.259). Results are illustrated in Figure 1. Controlling for the different times of day revealed no significant impact on the STD calibrations [F_(2, 100)_ = 0.856, *p* = 0.428].

### 3.2 Between-sessions correlations in calibration performance

**Figure 2:**
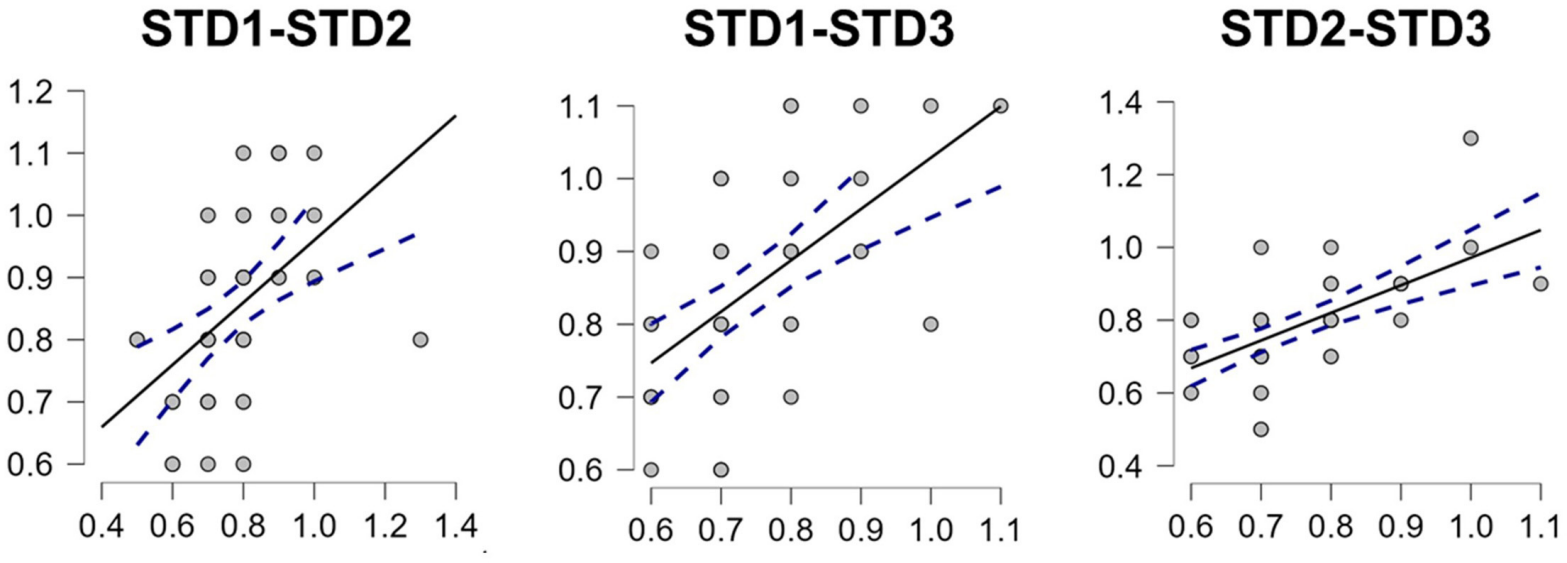
Scatterplots of the Mean STD correlations for calibration sessions 1 and 2 (rho = 0.62, ***p < 0.001), for sessions 1 and 3 (rho = 0.558, ***p < 0.001), and for sessions 2 and 3 (rho = 0.612, ***p < 0.001).

Considering that the mean STDs attained across the 3 calibration phases of the TloadDback showed continued improvement (with significant improvement occurring between sessions 1 and 2), we next assessed whether these STDs correlated with one another to investigate the intra-subject fidelity of the measure. Correlation analyses were carried out using Spearman's rho to determine whether or not the mean STDs from each of the 3 sessions correlated with one another. Results, shown in Figure 2, point to significant correlations between the three measures returned from the calibration sessions (all ps < 0.001). Additionally, an Intraclass Correlation (ICC) analysis was run, implementing the criteria of (1) the same fixed set of tests/raters as well as (2) the data being analyzed by mean values. The ICC returned a Cronbach's α of 0.78, supporting a significant correlation among the mean scores.

### 3.3 Verifying the distribution of change in STD between calibrations 1 and 2

**Figure 3:**
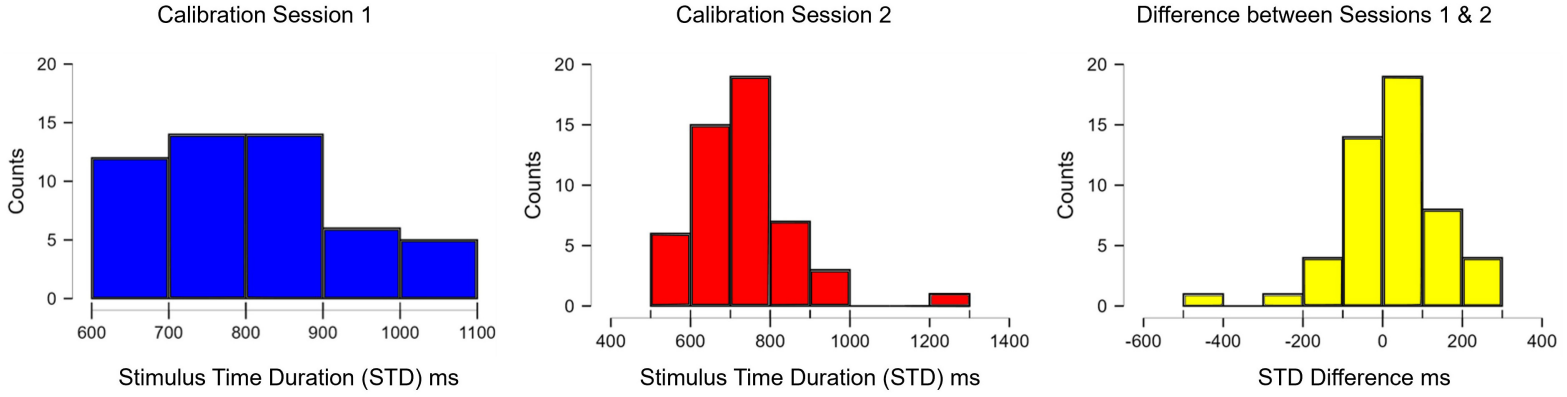
Frequency distribution of the STDs for calibration sessions 1 and 2 and the difference scores between both sessions. Positive scores reflect faster times or decreases in STDs, whereas negative scores show slower times or increases in the STDs.

A significant difference in calibrated STDs was found between sessions 1 and 2. In addition to calculating the mean difference score mentioned above, a frequency distribution was conducted to determine how much change occurred as well as for how many participants. In Figure 3, the majority of participants (*n* = 19, 37%) are shown to decrease by 100 ms, while the second majority (*n* = 14, 27%) are shown to have no change at all. That being said, ~64% of participants either stayed the same or improved by 100 ms, 16% improved by 200 ms, and 8% by 300 ms. Additionally, 8% showed an increase in STD by 100 ms and 2 participants (4%) showed decreases beyond 100 ms. Additionally, the number of blocks completed during session 1 showed a significant correlation with the difference between STDs from sessions 1 and 2 (rho = −0.608, *p* < 0.001). The same correlation was found for the number of blocks in session 2 and the difference in STDs between sessions 2 and 3 (rho = 0.502, *p* < 0.001). This shows that the higher the number of blocks completed in the preceding calibration led to a smaller amount of improvement between the two sessions (see Supplementary Figure S1).

### 3.4 Influences from control variables

In light of the significant differences between mean values from the TloadDback calibrations, we first planned to determine whether the 3 nights were similar or different. For each measure from the St. Mary' Sleep Quality Questionnaire (number of wakeups, number of hours of sleep, and quality of sleep), a Friedman's Non-parametric Repeated Measures ANOVA was conducted. All *p-values* for the 3 nights' sleep assessments did not reach significance (all ps > 0.05; see Supplementary Table S1). We additionally conducted correlation analyses with the STDs and the 3 items analyzed from the St. Mary' Sleep Quality Questionnaire for all of the 51 participants. A subset of participants (*n* = 31) also completed questionnaires on subjective sleepiness (KSS) and fatigue (VASf) at the beginning of each of the calibration sessions. Without correcting for multiple comparisons, the correlation matrices returned no significant correlations between any of the STDs and any of the other measures (all ps > 0.05). Correlation analyses can be found in Supplementary Table S2.

## 4 Discussion

The TloadDback procedure for identifying and implementing an individualized cognitive load through an optimal stimulus time duration (STD) calibrated for each participant relies heavily on the assumption that the STD achieved on 1 day during the calibration phase of this task is a reliable measure for individualized fatigue induction on another day. This task also relies heavily on the availability of attentional resources in order to perform effectively. Given that attentional resources can be impacted by natural changes in different factors, such as time of day (Brose et al., 2012; Matthews et al., 2017), sleep, sleepiness, and Fatigue (Mullins et al., 2014; Spruyt et al., 2019; Tomei et al., 2006; Walker and Trick, 2018), it is possible that performance on 1 day for this task might not be as reliable as performance on another day. The primary aim of this study was thus to determine how reliable the STDs are if they are attained at different times on different days while accounting for the different impacting factors.

With all things considered by the design in this study (time of day, quality of sleep, number of hours of sleep, number of wake ups, sleepiness, and fatigue), it appears that the only significant change found was a mean decrease in STDs between calibration sessions 1 and 2. Despite any differences in time of day, or the previous night's sleep or pre-task subjective assessments, participants improved their performance speed. In other words, participants continued to get faster between sessions 1 and 2. These findings could suggest that a second calibration would facilitate a better Stimulus-Response Mapping (Pfeuffer et al., 2018; Wickens et al., 2013) and that motor learning incrementally improves as well (Kimura and Nakano, 2019). This implies that a single calibration reports a slightly sub-maximal cognitive load because there is still room for some improvement. If such is the case, then the STD calculated to induce the maximal cognitive load after only 1 calibration might not be representative of the true maximal cognitive load that an individual can manage efficiently. In other words, participants completing the TloadDback after only 1 calibration are not being pushed to their true maximal cognitive load limit. This might also mean that by inducing CF after just one calibration, it might not develop so easily. However, while improvement in mean performance is observed between sessions 1 and 2, not all participants showed improvement. This indicates that compensation procedures that would decrease the STD of the first calibration by a constant (e.g., 100 ms), to compensate for the learning effect would not be fully appropriate for all participants. As shown above, because not all participants showed an improvement, these participants would be in a condition that supersedes their maximum capacity. The fact that all performances across the 3 calibrations were strongly correlated suggests that the calibration speeds for the STDs are rather reliable measures and that the inter-individual differences in performance are comparable between sessions. It is worth considering the accumulative gains across the 2 calibration sessions. The results show that between sessions 1 and 2, participants were, on average, 70 ms faster. Taking the format of the TloadDback into consideration (100 ms increments during the calibration phase) in conjunction with the calibration results, participants only gain on average less than one increase in cognitive load. This means that while participants might not be pushed to their exact maximal cognitive load, they are reliably pushed close to it. Furthermore, it was shown that the number of blocks in a single calibration significantly correlates with how much improvement is possible. An increased number of blocks in a single calibration means that there is continued learning during the session. This suggests that improvement is less likely. There may also be a ceiling effect in terms of performance improvement. As the presentation speed increases, the response window becomes increasingly smaller, making improvement much more difficult. For studies that have previously or are currently using the single calibration method, this small difference for the vast majority of participants should have placed or place participants in a near maximal cognitive load condition. As this condition is determined to be later used for inducing cognitive fatigue, a near maximal cognitive load condition should be sufficientnt to induce cognitive fatigue as shown in the seminal paper of Borragán et al. (2017b). Therefore, it should not have significantly affected the outcomes or interpretations of previous results using the TloadDback with a single calibration.

Regarding the ramifications for the TloadDback, it seems that researchers should decide whether it is reasonable to continue with a single calibration measure for a slightly sub-maximal cognitive load for fatigue induction or whether a second calibration session is conducted to determine a more accurate maximal cognitive load condition. With this in mind, implementing a truer maximum cognitive load condition could be attained by decreasing the STD by an additional 50 ms from the STD obtained after one calibration session. This would close the gap in the differences we found in our results for some participants and put them in a condition closer to their true maximal cognitive load. However, as not all participants showed an increase (37% remained the same or slower), there would be a risk of putting a participant in a condition beyond their maximum capacity. Notably, depending on the time and resources available, it could be worth investing in a second calibration session for assessing cognitive load.

Future research aiming to fine tune the implementation of this task or the understanding of cognitive fatigue could aim at discerning performance differences during the fatigue induction session as well as the changes in fatigue, from before to after the task, by looking at the different contributions from one vs. multiple calibration phases. Another factor, not considered in this study that would be worth investigating would be individual motivation levels before and after the task, especially in conjunction with multiple calibration sessions.

## 5 Conclusion

The results from this study suggest that a second calibration session could likely lead to a faster STD and higher cognitive load condition for fatigue induction. However, 2 calibrations only resulted in an STD improvement that was on average 70 ms faster than the STD achieved from a single calibration phase. In addition, despite improvement, all STDs were significantly correlated, suggesting a rather reliable measure. Conservatively speaking and given their reliability among the measures, the STD from the single calibration phase is likely enough to ensure an individualized cognitive load and a significant cognitive fatigue induction that accounts for inter-individual differences, as found in previous studies (Apreutesei and Cressman, 2024; Boaz-Curry, 2018; Borragán, 2018; Borragán et al., 2019; Charonitis, 2021; Guillemin et al., 2025, 2022; Holgado et al., 2023; Jacquet et al., 2021a,b; Pickering et al., 2024; Salomone et al., 2021; Smalle et al., 2021; Wierzchon and Derda, 2019). Nonetheless, it would be advisable to implement a second calibration in the protocol. Moving forward, researchers need to decide whether or not they need to conduct a second calibration sessions to better answer research questions.

## 6 Limitations

The current study did not take age or sex into consideration as variables that might influence outcomes of calibration phases. Ideally, a further look into such influences might prove beneficial in understanding the reliability and stability of the STD obtained from a single calibration phase. Future research could consider these factors.

## Data Availability

The datasets presented in this study can be found in online repositories. The names of the repository/repositories and accession number(s) can be found below: https://osf.io/8rmqg/.
