# Supplementary material for: Is a single calibration for the TloadDback cognitive fatigue induction task reliable?
Source: Front Psychol. 2025 Jul 24;16:1561819. doi: 10.3389/fpsyg.2025.1561819 (PMC12330871; doi:10.3389/fpsyg.2025.1561819)
Supplement: Supplementary file 1 [file Supplementary_file_1.docx]

# Supplementary Material


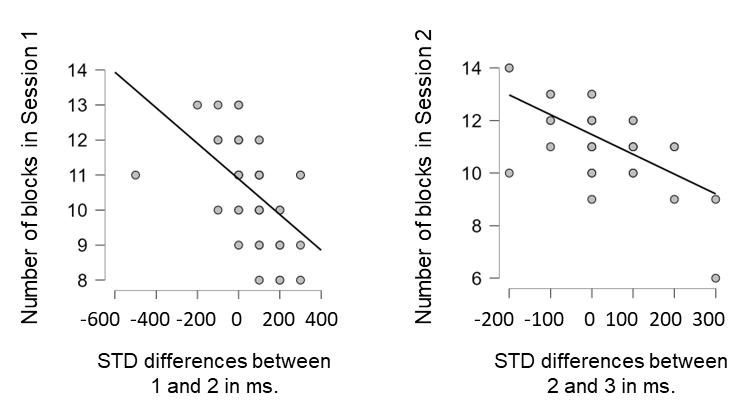


Figure S1. Scatterplot of the number of blocks against the differences in STDs. The number of blocks in session 1 shows a significant correlation with difference found between the STDs in sessions 1 and 2 (rho = -0.608, p < 0.001***). The same correlation is found for the number of blocks in session 2 and difference between sessions 2 and 3 (rho = -0.502, p < 0.001***). This suggests that the higher the number of blocks, the lower the likelihood for an improvement in the following session. p < 0.05*, p < 0.01**, p < 0.001***.

Descriptives

| **Variable** | **Valid** | **Median** | **Mean** | **Std. Dev.** | **Shap.-Wilk**  **P-value** | **Min** | **Max** |
| --- | --- | --- | --- | --- | --- | --- | --- |
| STD1 | 51 | 0.8 | 0.849 | 0.138 | 0.01** | 0.6 | 1.1 |
| STD2 | 51 | 0.8 | 0.778 | 0.135 | <0.001*** | 0.5 | 1.3 |
| STD3 | 51 | 0.7 | 0.745 | 0.11 | <0.001*** | 0.6 | 1.1 |
| QoS1 | 51 | 4 | 4.431 | 0.985 | <0.001*** | 3 | 6 |
| QoS2 | 51 | 4 | 4.118 | 1.032 | <0.001*** | 1 | 6 |
| QoS3 | 51 | 4 | 4.353 | 0.913 | <0.001*** | 2 | 6 |
| HrsSlp1 | 51 | 7.5 | 7.333 | 1.123 | 0.439 | 5 | 9.67 |
| HrsSlp2 | 51 | 7.5 | 7.424 | 1.304 | 0.775 | 3.67 | 10.5 |
| HrsSlp3 | 51 | 7.67 | 7.423 | 1.491 | 0.002** | 4 | 13 |
| WKUp1 | 51 | 1 | 1.627 | 1.562 | <0.001*** | 0 | 7 |
| WkUp2 | 51 | 1 | 1.647 | 1.671 | <0.001*** | 0 | 7 |
| WkUp3 | 51 | 1 | 1.667 | 1.751 | <0.001*** | 0 | 7 |
| KSS1 | 31 | 4 | 4.039 | 1.574 | 0.005** | 1 | 7 |
| KSS2 | 31 | 3 | 4.097 | 1.758 | 0.009** | 1 | 7 |
| KSS3 | 31 | 3 | 3.548 | 2.11 | 0.003** | 1 | 8 |
| VASf1 | 31 | 3.64 | 4.15 | 2.063 | 0.355 | 0.17 | 7.83 |
| VASf2 | 31 | 4.13 | 4.404 | 1.827 | 0.875 | 0.83 | 0.25 |
| VASf3 | 31 | 2.98 | 3.437 | 2.088 | 0.031* | 0.25 | 7.93 |

Table S1. Descriptive statistics for Stimulus Time Durations (STD), Quality of Sleep (QoS), the number of hours of sleep (HrsSlp), the number of wakeups (WkUp), the Karolinska Sleepiness Scale scores (KSS) and the Visual Analog Scale for fatigue (VASf) for each of the Calibration Sessions (1, 2 and 3). The majority of the results violated normality according to the Shapiro-Wilk test.

*≤ 0.05 **≤0.01 ***≤0.001.

STD Correlation Analyses with Control Variables

Table S2. Correlation Matrix for the mean STDs of each Calibration Session with the variables for the Quality of Sleep (QoS), the number of hours of sleep (HrsSlp), the number of wakeups (WkUp), the Karolinska Sleepiness Scale (KSS) and the Visual Analog of fatigue scale (VASf). Each variable is marked as 1, 2 or 3 which corresponds to its calibration session. Significant values are noted with asterisks. * ≤ 0.05 ** ≤ 0.01 *** ≤ 0.001
